# Cohesin in Oocytes—Tough Enough for Mammalian Meiosis?

**DOI:** 10.3390/genes1030495

**Published:** 2010-12-13

**Authors:** Ekaterina Revenkova, Caroline Adelfalk, Rolf Jessberger

**Affiliations:** 1 Department of Developmental and Regenerative Biology, Mount Sinai School of Medicine, One Gustave L. Levy Place, Box 1020, New York, NY 10029, USA; E-Mail: ekaterina.revenkova@mssm.edu; 2 Institute of Physiological Chemistry, Faculty of Medicine Carl Gustav Carus, Dresden University of Technology, Fiedlerstr. 42, MTZ, D-01307 Dresden, Germany; E-Mail: caroline.adelfalk@tu-dresden.de

**Keywords:** oocytes, cohesion, aneuploidies

## Abstract

Sister chromatid cohesion is essential for cell division. During meiosis, it is also required for proper synapsis of pairs of sister chromatids and for chiasma formation and maintenance. Since mammalian oocytes remain arrested in late prophase for a very long period—up to five decades in humans—the preservation of cohesion throughout this period is a formidable challenge. Mouse models with cohesin deficiencies and aging wild-type mice showed that this challenge is not fully met: cohesion weakens and deteriorates with increasing age. These recent findings have highly significant implications for our comprehension of the genesis of aneuploidies.

Is there anything more demanding for cohesin than mammalian oocyte meiosis? Probably not, since not only does meiosis-specific cohesin need to be established at the onset of meiosis and to be adjusted to support pairing of homologous chromosomes and their recombination, and not only does it need to be released in a carefully orchestrated two-step mechanism, it also needs to be maintained for a long period of time after birth of the female organism. In humans, oocytes arrested in late prophase—the dictyate arrest stage—may remain quiescent for about five decades from birth to the end of reproductive life. During this long period, cohesion between sister chromatids needs to be sustained to allow proper segregation of chromosomes in both meiotic divisions. 

The basic process of meiosis is described in several articles in this volume and shall not be re-described here. The particularities of cohesin, however, will be discussed in detail.

Sister chromatid cohesion is achieved by the essential four-subunit cohesin complex (for reviews see [1,2,3,4]). In somatic cells the complex consists of two SMC proteins, SMC1α and SMC3, as well as the kleisin RAD21 (SCC1) and SA1 or SA2 as SCC3-type proteins. RAD21 closes the cohesin ring that is pre-formed by the V-shaped SMC1/3 dimer. SCC3-type proteins were hypothesized to link two of such tripartite rings thereby forming a “handcuff”-like structure [5]. The precise function of SA1 and SA2 remains to be described, but distinct roles of SA1 and SA2 in supporting cohesion at telomeres and centromeres, respectively, were observed in HeLa cells [6].

In vertebrate meiocytes, meiosis-specific cohesin proteins replace the “canonical” ones to a large extent. The meiotic kleisin REC8 substitutes for RAD21, STAG3 replaces SA1/SA2, and SMC1β replaces SMC1α. Yet, at the initial stages of meiosis, the cohesin complex featuring SMC1α is still associated with chromosomes [7]. The SMC1β-based cohesin complex is present in mouse spermatocytes and oocytes throughout meiosis up to the metaphase/anaphase II transition [8]. It appears as if SMC1α and SMC1β each form several different complexes with different non-SMC subunits. For example, SMC1β can be immunoprecipitated from testis nuclear extracts with REC8 or with RAD21 [9]. The functional specialization of these distinct complexes is not known.

SMC1β deficiency causes complete loss of sister chromatid cohesion in metaphase II as observed in *Smc1*β*^-/-^* oocytes, which—unlike *Smc1*β*^-/-^* spermatocytes that die in mid-pachytene—survive up to the metaphase II/anaphase II transition [9]. Thus, SMC1β is essential for continuous meiotic sister chromatid cohesion, which cannot be sustained by endogenous SMC1α alone. Axial elements and synaptonemal complexes are about half as long in prophase I *Smc1*β*^-/-^* oocytes and spermatocytes compared to wild-type meiocytes. *Smc1*β*^-/-^* prophase I oocytes and spermatocytes also show incomplete synapsis, reduced numbers of MLH1 foci indicative of recombination sites, and aberrant telomere structures [9,10,11]. Importantly, SMC1β deficiency in oocytes causes loss of chiasmata resulting in a dramatic increase in the frequency of univalents, *i.e.*, pairs of sister chromatids not associated with the homologous pair. In metaphase I oocytes from two month old *Smc1*β*^-/-^* mice, 62% of chromosomes showed as univalents. In the majority of the homologs that remained in the bivalent configuration, chiasmata were located in chromosome segments most distal from the centromeres in contrast to evenly distributed MLH1 foci observed in pachytene. 

These data suggested that without SMC1β-mediated sister chromatid cohesion, chiasmata cannot be maintained. There may be several explanations, but the most straightforward hypothesis is that in the mutant, chiasmata initially properly positioned at the crossover sites gradually shifted towards the ends of chromosomes and eventually “slipped” off telomeres. Indeed, the shorter mouse chromosomes show the highest frequency of chiasma loss, consistent with this hypothesis. Another striking effect of cohesin deficiency in the *Smc1*β*^-/-^* oocytes is a highly elevated incidence of single chromatids. Both loss of chiasmata and premature separation of sister chromatids were exacerbated with age [12]. With these phenotypes, the *SMC1*β deficient mouse was considered a model that in certain, albeit limited, respects reflects the age-dependent increase in aneuploidies seen in humans [13,14,15]. In humans, a rapid increase of trisomic fetuses is observed with increasing female age, particularly above 35 years [16]. Most of these aneuploidies originate from errors in oocyte meiosis I or meiosis II [17]. Loss of cohesion with increasing age, like in female *Smc1*β*^-/-^* mice, may contribute to increased aneuploidy in ageing human oocytes.

Rec8 deficient mice show a distinct phenotype [18,19]. Besides deficiencies in sister chromatid cohesion and in synapsis—which occurs between sister chromatids instead of homologs—they are sterile. Oocytes and ovarian follicles were absent already in day 5 neonates, and typical pachytene oocytes were not found in day 16.5 to 18.5 embryonic ovaries. 

Recently, several studies appeared that shed new light on the role of cohesin in preventing aneuploidies. These reports together very significantly extend our knowledge on the status of cohesin in mammalian ocytes, how cohesin acts, and when and where cohesin is present and required.

Following an initial study limited to prophase I [20], Garcia-Cruz *et al.* [21] analyzed human oocytes throughout meiosis for the presence and localization of individual cohesin proteins, including the three meiosis-specific ones, REC8, STAG3, and SMC1β, as well as SMC3. These four cohesins co-localize with SYCP3, a component of the axial elements and of the lateral elements of the synaptonemal complex (SC). Upon full synapsis, the cohesins also co-localize with the SC central element protein SYCP1. On metaphase I chromosomes, the cohesins localize to centromeres, along chromosome arms, and at each side of chiasmata, but not directly on top of chiasmata. From anaphase I to metaphase II, the cohesins were detected at the centromeres only, and disappeared thereafter. SMC1α and RAD21 were not included in this study, and thus there localization in human oocytes remained unclear. These results are largely in agreement with results obtained from mouse oocytes. Garcia-Cruz *et al.* quantified *Smc1*β transcripts from germinal vesicle stage (GV) oocytes from women of various age ranging from 19 to 43 years, but high variability between samples prevented a clear conclusion with regard to a correlation with age. In mice, *Smc1*β transcripts, measured by real-time PCR, remain present in oocytes at least up to six months of age, albeit at relative amounts that are less than 10% of those found in pachytene oocytes [12].

To clarify whether *Smc1*α is expressed in oocytes after birth, we evaluated the relative amount of *Smc1*α mRNA by real-time PCR in oocytes collected from mouse embryos, new-born, pre-pubertal, and adult animals (Figure 1A). Initially expression of *Smc1*α was low from day 16 post coitum until the newborn stage. However, the expression of *Smc1*α strongly increased in one month-old and older animals. For example, expression increased more than 8-fold in growing two month-old oocytes compared to the newborn. One plausible explanation of such an expression pattern is that growing oocytes accumulate a storage pool of *Smc1*α mRNA for future synthesis of mitotic cohesin necessary during the first, rapid embryonic cell divisions.

**Figure 1:**
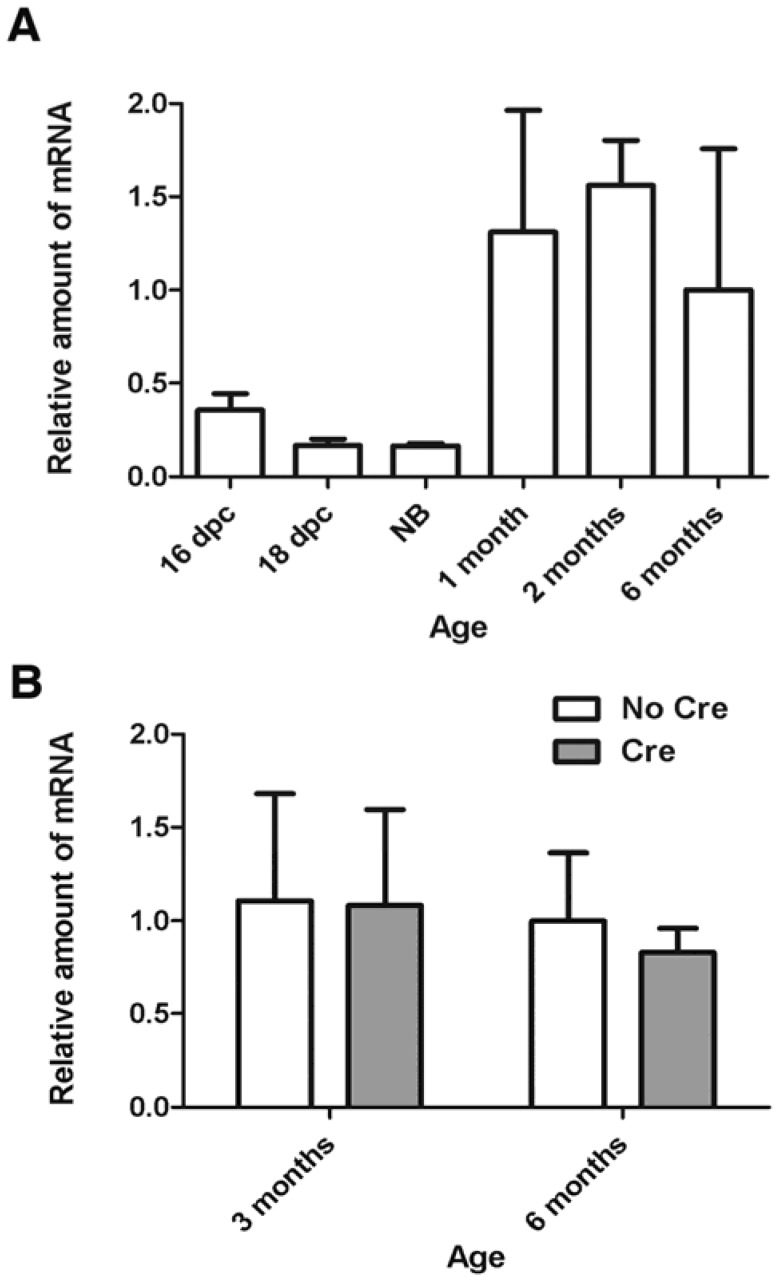
Expression of the *Smc1*α gene in mouse oocytes. Relative amount of *Smc1*α mRNA was measured by quantitative real-time PCR as described earlier [10,20]. The error bars represent standard deviations. (**A**) Relative amount of *Smc1*α transcript increases in growing wild-type oocytes. Dpc-days post coitum, NB-newborn; (**B**) Cre-mediated inactivation of *Smc1*β in *Smc1*β*^fl/-^* mice does not affect the amount of *Smc1*α mRNA in germinal-vesicle stage oocytes. Analysis of variance by two-way ANOVA showed that effects of genotype or age were not significant (*p* > 0.2).

Another possibility is that the increased amount SMC1α-type cohesin complex is necessary for regulation of gene expression in oocytes undergoing maturation. The role of cohesins in regulation of gene expression has been demonstrated in various somatic mammalian cell lines and in embryonic stem cells [22,23,24,25,26], but has to be elucidated in germ cells. Importantly, in the *Smc1*β*^-/-^* mice the pattern of the *Smc1*α gene expression did not change, and *Smc1*α could not compensate for the absence of functional *Smc1*β [12].

If slow deterioration of cohesin is a major cause for mis-segregation in oocytes, one may expect to see a decrease in cohesion with age even in wild-type mice. A very recent report by Chiang *et al.* [27] shows that in 16 to 19 month old mouse oocytes metaphase I and metaphase II sister kinetochores are less tightly connected, *i.e.*, they are farther apart, indicative of weakened cohesion. Concomitantly, the amount of chromosome-associated REC8 cohesin is significantly decreased in old oocytes, which give rise to aneuploid eggs at a significantly higher rate than young oocytes. REC8 disappears from chromosomes gradually as mice age, and only after it drops below 10% of the level found in three-month-old mice, the segregation error rate begins to steeply rise as revealed by live imaging of oocytes undergoing anaphase I. Reduced presence of REC8, STAG3, and SMC1β on chromosomes of aged senescence-accelerated mice, which show premature separation of sister chromatids, has also been observed earlier [28].

Similarly, Lister *et al.* [29] found loss of chiasmata and of REC8 from metaphase I chromosomes in aging wild-type mice. Lister *et al*. also examined colocalization of REC8 and DNA in GV oocytes and concluded that cohesion loss occurs while oocytes age when arrested in prophase I. In addition, the cohesin protector protein Sgo2 becomes depleted with increasing age of the mice, suggesting increasing vulnerability of cohesin to proteolysis.

Why do aging chromosomes lose cohesin? A certain minimum amount of cohesin associated with a chromosome is apparently necessary to provide cohesion and to prevent aneuploidy. How and when is the initial amount of cohesin produced, loaded onto chromosomes, and maintained? And why do cohesin and Sgo2 slowly disappear? The stability of cohesin molecules remains elusive. If one assumes that sister chromatid cohesion relies solely on the cohesin loaded at the initial stages of meiosis, slow protein degradation may deplete cohesin from chromosomes. A single endoproteolytic cleavage of the kleisin or in any other part of the cohesin ring is in principle sufficient to remove the ring from chromosomes [3]. On the other hand, one cannot *a priori* exclude that degraded cohesin is successfully replaced by newly loaded molecules. This re-loading process may lose efficiency in older mice. Reloading would very likely require *de novo* synthesis of cohesin proteins during prophase arrest.

Thus, an important question relates to the requirement for cohesin during and after the dictyate stage: is continuous expression of cohesin required to maintain cohesion and prevent aneuploidies? This question was addressed by constructing a mouse strain that has loxP sites inserted in the *Smc1*β gene to create a conditional *Smc1*β*^fl^* allele [30]. After the expression of Cre recombinase driven by the *Gdf9* promoter starts in oocytes within hours after birth, the *Smc1*β gene is no longer capable of producing functional transcripts, which disappear between day 4 and 6 post partum. Thus, oocytes in these *Smc1*β*^fl/fl^* GDF9-Cre (“F1”) mice lack any source of newly synthesized SMC1β-type cohesin from dictyate arrest onwards. Quite surprisingly, these mice stay fully fertile and their oocytes do not show any impairment of sister chromatid cohesion and chiasmata maintenance and positioning up to eight months of age. Notably, the absence of *Smc1*β expression did not cause an increase in *Smc1*α transcript levels (Figure 1B). All progeny (“F2”) of the F1 mice lacked *Smc1*β expression and were sterile, demonstrating the high efficiency of Cre-mediated excision. The F2 phenotype was identical to that of the regular *Smc1*β*^-/-^* mice.

Since continuous SMC1β cohesin expression during the dictyate stage is not necessary for fertility, cohesion and chiasmata maintenance, one may ask whether cohesin is indeed still present on metaphase I chromosomes after the *Smc1*β exon was excised shortly after birth. We determined the presence of STAG3, SMC3, and SMC1β on oocyte metaphase I chromosomes (Figure 2). All these cohesins were seen associated with metaphase chromosomes of F1 oocytes in a pattern very similar if not identical to that of wild-type mice. Most staining was observed at the centromeres. In contrast, *Smc1*β*^-/-^* oocytes showed loss of STAG3 and SMC3, indicating complete absence of cohesin and the occasional occurrence of univalents, as demonstrated before [12].

**Figure 2:**
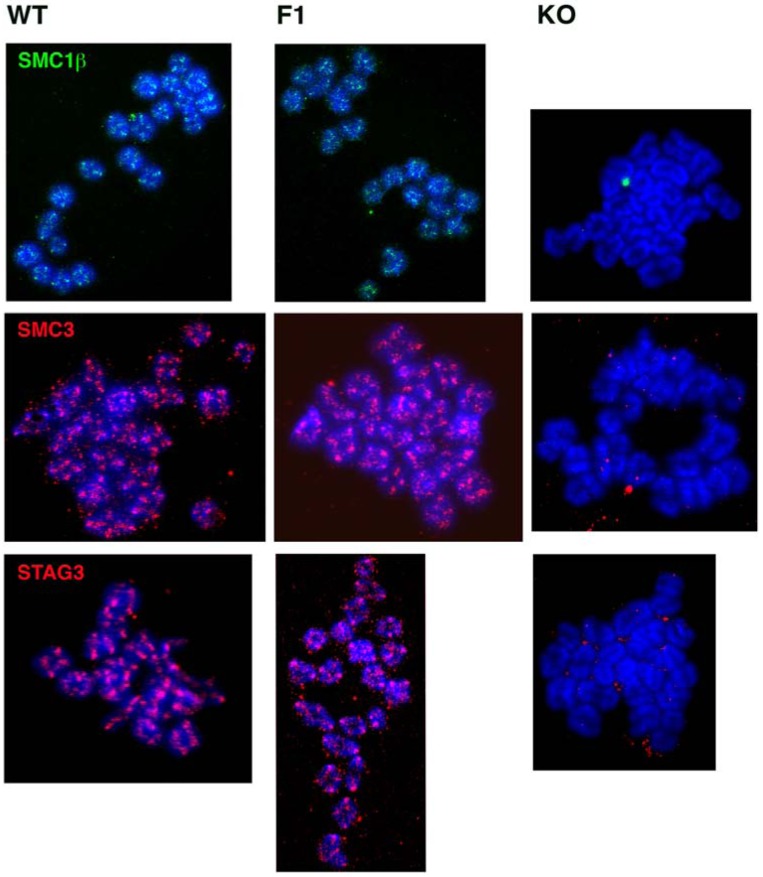
Immunofluorescence staining of cohesins SMC1β (green), SMC3 (red), and STAG3 (red) on metaphase I chromosomes of oocytes from wild-type (WT), *Smc1*β*^fl/fl^ GDF9-Cre* (F1), and *Smc1*β*^-/-^* (KO) mice of two-to-three months of age. Chromosomes are stained by DAPI (blue).

A study by the Nasmyth lab [31] adds another very important insight, as it addresses questions of REC8 function in oocytes and the question of whether REC8 can be reloaded in growing oocytes, and thus whether there is turnover of REC8 cohesin. Tachibana-Konwalski *et al.* engineered mouse strains expressing REC8 and SCC1 with target sites for TEV protease. Cleavage by TEV could efficiently destruct REC8- or SCC1- cohesins after *Rec8^TEV/TEV^* or *Scc1^TEV/TEV^* oocytes were microinjected with TEV mRNA. TEV-mediated cleavage of REC8 in *Rec8^TEV/TEV^* oocytes was sufficient for chiasma resolution even when securin degradation was inhibited by overexpression of MAD2. Furthermore, TEV mRNA injected into *Rec8^TEV/TEV^* GV oocytes caused complete separation of sister chromatids. On the contrary, TEV expression in *Scc1^TEVMyc/TEVMyc^* oocytes did not affect bivalent structure. Additionally, TEV expression in meiosis II arrested oocytes led to disjunction of sister centromeres in *Rec8^TEV/TEV^* oocytes, but did not affect cohesion between sister centromeres in *Scc1^TEVMyc/TEVMyc^* oocytes. Thus, the authors elegantly demonstrated that REC8-containing cohesin is necessary and sufficient for both arm and centromere cohesion in oocytes. Then what is the function of SCC1, which is abundant in oocytes? TEV-mediated destruction of SCC1 in zygotes caused loss of sister chromatid cohesion in metaphase of the first embryonic division, but cleavage of REC8 did not have any effect, demonstrating that a switch from meiotic REC8-dependent and SCC1-independent cohesion in oocytes to REC8-independent and SCC1-dependent cohesion in the embryo occurs in the zygote. A requirement for a rapid switch from meiotic to mitotic cohesion may also explain the accumulation of the *Smc1*α transcripts in growing oocytes (Figure 1B).

Tachibana-Konwalski went further and added a stage-specific REC8 expression cassette to their TEV system, which allowed complementation of cleavable REC8-TEV with uncleavable REC8 either at early meiosis or in primary follicles. Uncleavable Rec8 could prevent cohesion destruction by TEV only if present during the initial meiotic cohesion establishment, but expression in growing oocytes had no effect. This strongly supports the hypothesis that cohesins involved in meiotic sister chromatid cohesion are not replaced or at least not sufficiently by newly synthesized molecules during oocyte arrest.

Together, there is now sound evidence from different experimental approaches, which clearly supports the hypothesis of cohesin deterioration as a leading cause for age-dependent oocyte aneuploidies at least in mice. Figure 3 summarizes key insights related to aneuploidies associated with diminished cohesion. 

**Figure 3:**
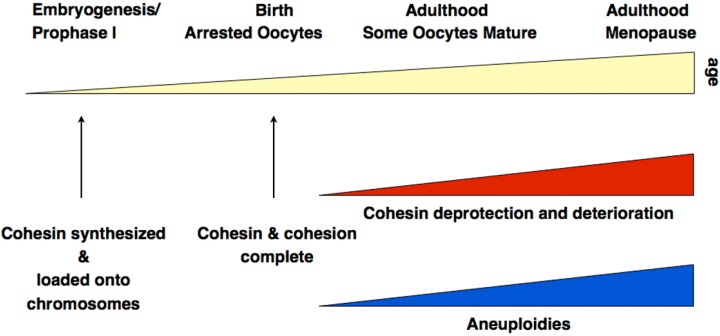
Summary of cohesin-related processes relevant for aneuploidies. The precise rates of increase of cohesin loss and of aneuploidies, and their timing remain unknown and are only shown as an approximate correlation.

There are, obviously, many important questions still to be solved. Whether the results obtained in mouse experiments can indeed explain some of the stunning increase in aneuploidies seen in embryos of aging human mothers needs to be shown. The mechanisms and timing of loading of the various cohesin complexes onto germ cell chromosomes is not yet clear, and the full spectrum of functions of cohesins in meiocytes has certainly not yet been described. Why do mammals form four or perhaps more different types of cohesin complexes in meiocytes and which specific functions do these complexes have, and why did a second SMC1-type protein, SMC1β, evolve in vertebrates? 
